# Experiences, attitudes, and perceptions of caregivers of individuals with treatment-resistant schizophrenia: a qualitative study

**DOI:** 10.1186/s12888-018-1833-5

**Published:** 2018-08-13

**Authors:** Cecilia Brain, Steven Kymes, Dana B. DiBenedetti, Thomas Brevig, Dawn I. Velligan

**Affiliations:** 1 H. Lundbeck A/S, Valby, Denmark; 2 Lundbeck US, Deerfield, IL USA; 3 RTI Health Solutions, Research Triangle Park, 27709 NC USA; 4 University of Texas Health Science Center, San Antonio, TX USA

**Keywords:** Treatment resistance, Non-response, Antipsychotic, Caregiver burden, Focus groups, Persistent symptoms, Impact

## Abstract

**Background:**

Treatment-resistant schizophrenia (TRS) affects about one-third of individuals with schizophrenia. People with TRS do not experience sustained symptom relief and at the same time have the most severe disease-related disability and associated costs among individuals with severe mental disorders. Like caregivers of people with treatment-responsive schizophrenia, caregivers of individuals with TRS experience the disease burden along with their care recipients; however, for those providing care for individuals with TRS, the stress of the burden is unrelenting due to uncontrolled symptoms and a lack of effective treatment options. The objective of this study is to better understand the burden of TRS from the caregiver perspective and to explore their perception of available treatments.

**Methods:**

Eight focus groups with non-professional, informal caregivers of individuals with TRS were conducted in 5 US locations. TRS was defined as failure of ≥2 antipsychotics and persistent moderate-to-severe positive symptoms of schizophrenia, per caregiver report.

**Results:**

The 27 caregivers reported an average of 37 h/week providing direct care, and 21 reported being on call “24/7.” Caregivers commonly reported that their care recipients exhibited symptoms of auditory hallucinations (89%), agitation/irritability/hostility (81%), suspiciousness (78%), tangentiality (74%), and cognitive impairment (74%); 70% of caregivers ranked suspiciousness/persecution as the most challenging symptom category. Caring for an individual with TRS impacted many caregivers’ finances, career prospects, social relationships, and sense of freedom. Additionally, multiple medication failures led to a sense of hopelessness for many caregivers.

**Conclusions:**

Persistent positive symptoms caused significant perceived burden, feelings of being overwhelmed and having no relief, and substantial negative impacts on caregivers’ emotional and physical health. To address these substantial unmet needs, policy makers should be aware of the need for practical, social, and emotional support for these caregivers and their families. Additionally, new treatment options for TRS should be developed.

## Background

The core symptoms of schizophrenia include positive symptoms (i.e., hallucinations, delusions, disorganized speech, or suspiciousness/persecution), negative symptoms (e.g., affective flattening, alogia, avolition, and anhedonia), and other associated symptoms (e.g., cognitive impairment) [1]. Atypical and typical antipsychotic (AP) medications, the cornerstone of treatment for schizophrenia, target the dopamine D_2_ receptor to reduce positive symptoms of schizophrenia [2], the key focus of AP treatment [3]. Schizophrenia that is non-responsive to treatment with an atypical or typical antipsychotic (AP), resulting in persistent positive symptoms [4, 5], may constitute a distinct subtype of the disease requiring a different treatment approach [6–8]. Treatment-resistant schizophrenia (TRS), clinically defined as failure to respond to two trials of APs of adequate dose and duration, affects about one-third of individuals with schizophrenia [5]. Treatment guidelines recommend the use of clozapine after 2 AP failures [9–11]. However, clozapine initiation is typically delayed in favor of increased dosage of the current AP, switching to other APs, or combination therapy (AP polypharmacy) [4, 12]. The reason for resorting to these non–evidence-based treatment options over clozapine is not well understood, but safety, tolerability, and monitoring issues may relegate it to later-line use. Caregiver engagement through shared-decision making has been shown to increase knowledge about treatments and improve perception and drug attitude towards antipsychotics by supporting informed decisions by the person with schizophrenia [13–15].

TRS is associated with poor functional outcomes. For example, individuals with TRS exhibit significantly lower psychosocial functioning than treatment-responsive patients and lower cognitive performance than patients with other serious psychiatric conditions [16]. Individuals with TRS are at increased risk of unemployment, homelessness, aggression, imprisonment, substance abuse, violent victimization, and suicide [1, 16–19]. The economic burden of TRS is also significant, driven by increased health care utilization due to longer and more frequent hospitalizations and social service costs [18]. Individuals with TRS are also less likely than individuals with treatment-responsive schizophrenia to live independently [16, 18]. Arguably, TRS poses the greatest disability of all mental illnesses [10, 16]. This burden places a particular challenge on those providing informal, unpaid care to people with TRS. Individuals with schizophrenia may be highly reliant on caregivers to provide assistance with their daily activities (e.g., managing treatment decisions and medications, finances, transportation, meals, and housework) [20]. Additionally, unlike individuals with treatment-responsive schizophrenia, who experience cycles of remission and relapse of symptoms [21], individuals with TRS experience persistent positive symptoms, often with negative symptoms and/or cognitive dysfunction [9, 22], without periods of symptom remission. Thus, for their caregivers, the stress and burden of the disease persists without respite.

Previous research exploring the experiences of caregivers of individuals with schizophrenia and other psychotic disorders has found the objective and subjective burdens of providing informal care to be considerable [23–25]. Informal caregivers of individuals with severe mental illness report spending, on average, 22 h or more per week on care-related activities [25, 26]. In addition, half of caregivers report that their caregiving role has a negative impact on their mental health, and one-third report an impact on their physical health [25, 26]. Caregivers of adults with schizophrenia in particular experience impairments in the mental health and interference in their daily functioning and social functioning as a result of emotional problems [20].

However, the experiences specific to caregivers of individuals with TRS have not been previously studied, representing a significant gap in the literature. It is reasonable to expect that caring for people with persistent moderate-to-severe schizophrenia symptoms that remain despite several treatment trials places a more onerous burden on caregivers than that of caring for someone for whom medication has been effective and whose symptoms are mitigated by treatment. Thus, the objective of this qualitative study was to investigate the burdens on caregivers for persons with TRS, to understand their experiences and challenges, and to document their attitudes toward and perceptions of available treatments.

## Methods

### Study design and participants

Eight focus groups consisting of informal caregivers for persons with TRS who were currently being treated with one or more APs were conducted at facilities that specialize in recruiting participants for qualitative research in five US locations (Chicago, Illinois; Philadelphia, Pennsylvania; Raleigh, North Carolina; Los Angeles, California; and Phoenix, Arizona). The focus groups were led by two experienced qualitative researchers, including a licensed clinical psychologist with significant experience in schizophrenia (D.B. DiBenedetti). At least one representative from the study sponsor observed the focus groups behind a one-way mirror. The study was approved by RTI International’s institutional review board, and all caregivers provided written informed consent.

Medical recruiters at each qualitative facility recruited the participants by contacting members in their databases who had previously indicated providing informal caring for someone with schizophrenia and/or advertising on their internal websites. Potential participants were screened using a recruitment script.

Focus group participants were adult caregivers providing informal, unpaid care to an adult with TRS (according to caregiver report). Eligible caregivers must have been in a caregiving role for ≥1 year and currently spend, in a typical week, ≥4 h providing direct care and ≥ 20 h providing overall care (direct or being “on call”) to the individual with schizophrenia. The care recipient must have been, by caregiver report, age ≥ 18 years, have received a clinician-provided diagnosis of schizophrenia ≥1 year ago, have schizophrenia as the most recent diagnosis in the event of multiple psychiatric diagnoses (bipolar disorder or schizophrenia-related disorders, such as schizoaffective, schizophreniform, schizotypal personality, or brief psychotic disorder), currently be treated with and adherent to an AP medication (adherence defined as taking medication as prescribed ≥80% of the time), and have met the study criteria for TRS. TRS was defined as caregiver-reported failure of ≥2 AP medications of adequate dose for ≥6 weeks’ duration, with at least one being an atypical AP, and at least moderate severity in two or more core positive symptoms of schizophrenia.

Caregivers of persons with TRS who were currently on clozapine were excluded from the study. A rationale for this exclusion was that few individuals with schizophrenia are prescribed clozapine, and caregivers of individuals receiving clozapine might not represent the real-world experiences of those caring for individuals with TRS and persistent schizophrenia symptoms. In addition, the experiences of caregivers caring for the severely disabled subgroup of individuals with schizophrenia not responding to clozapine (i.e., with ultra-resistant disease) are likely to be different from TRS in general, and the goal of the study was to capture the experience of caregivers for persons with “typical” TRS.

### Focus group procedure

A semi-structured discussion guide was used during the focus groups. Clinical experts, patient representatives from National Alliance on Mental Illness and Mental Health America, and economic experts with experience in qualitative research reviewed and provided input on the guide before it was finalized. A standard line of questioning was included in the guide but was adjusted for each group based on participants’ understanding of the discussion topics and specific experiences. The guide included probe questions to better understand caregivers’ responses to the discussion questions. Each focus group lasted approximately 90 min and was audio-recorded and transcribed. The transcripts were reviewed for accuracy by an independent researcher.

Focus groups began with an open-ended discussion of participants’ responsibilities in caring for an individual with TRS, as well as caregivers’ role in ensuring that care recipients took their schizophrenia medication, caregivers’ involvement with health care providers, and any time spent “on call.” Discussion then focused on the persistent schizophrenia symptoms that care recipients experienced. Specifically, caregivers were given a checklist of positive symptoms (hallucinations, delusions, disorganized speech, suspiciousness/persecution), negative symptoms, and other symptoms based on the core and associated symptoms of schizophrenia included in the *Diagnostic and Statistical Manual of Mental Disorders, Fifth Edition* [1], the Positive and Negative Syndrome Scale [27], and the Brief Psychiatric Rating Scale [28]. All symptoms/behaviors were presented to caregivers in non-clinical terms (see Table 1). Caregivers selected the symptoms their care recipient reported or demonstrated, ranked which symptom categories were most challenging for them as caregivers, and shared with the group members the impact of these symptoms on themselves and the care recipient. Additional discussion focused on caregivers’ and care recipients’ experiences with AP medications, including perspectives on medication efficacy and attitudes towards repeated medication failures. Caregivers also described how their lives would be impacted if a medication would relieve the care recipients’ persistent symptoms.

**Table 1:** Symptoms/behaviors exhibited or reported by care recipients (*N* = 27)

DSM-5 Category and Symptom	Description Provided to Caregiver	Caregiver-Reported Frequency, *n* (%)
Hallucinations	Patient heard, saw, tasted, felt or smelled things that others did not experience	
Auditory hallucinations	Heard noises or voices or things that other people did not hear	24 (89)
Visual hallucinations	Saw things other people did not see	15 (56)
Tactile hallucinations	Felt things on the body other people did not feel or notice, for example people touching/hitting him/her; reported strange feelings underneath his/her skin	10 (37)
Olfactory hallucinations	Smelled things other people do not seem to smell	8 (30)
Delusions	Patient had unusual or odd beliefs or thoughts that other people did not understand	
Referential delusions (external stimuli directed toward patient)	Had beliefs that certain things in the environment, gestures, comments, etc. were directed towards him/her; reported experiences with mind reading, psychic forces, or fortune telling	19 (70)
Delusions of love	Had false beliefs that someone is in love with him/her	10 (37)
Nihilistic delusions	Strongly believed that a major catastrophe will occur	9 (33)
Somatic delusions	Had preoccupations with health, bodily function, organs	8 (30)
Delusions of grandeur	Had beliefs of exaggerated or extreme importance, wealth, power, or goodness (e.g., saving the world, ending poverty, stopping wars)	4 (15)
Delusions of grandeur	Had strong beliefs that he/she had extraordinary fame, wealth, gifts/talents, or abilities	3 (11)
Disorganized speech	Patient demonstrated unusual, disorganized or confused ways of speaking/thinking	
Tangentiality	Patient had trouble getting his/her point across when talking, often rambled or got off track	20 (74)
Derailment, loose associations	Patient frequently switched from one topic to another during a conversation	18 (67)
Incoherence, word salad	Others had trouble understanding/following what [patient] was saying	15 (56)
Incoherence	Patient’s speech did not make sense	12 (44)
Suspiciousness/persecution	Patient was overly suspicious or felt like he/she was being persecuted	
Suspiciousness	Did not trust or was suspicious of people	21 (78)
Paranoia	Felt like other people were watching or talking about him/her	19 (70)
Persecutory delusion	Believed that he/she is going to be harmed or harassed	14 (52)
Delusion of control	Felt that someone or something was controlling his/her ideas or thoughts	10 (37)
Negative symptoms		
Asociality	Withdrew from being around other people, family, or friends (e.g., did not make eye contact with others, not seem to enjoy being around others, spent a lot of time sitting or lying around much of the day)	19 (70)
Amotivation	Had difficulty starting and completing activities	18 (67)
Limited interest/avolition	Sit/lay around for long periods of time with limited interest in things	18 (67)
Anhedonia	Seemed to have less enjoyment or pleasure in things	16 (59)
Blunted affect, diminished emotional expression	Showed little emotion (feeling) or inappropriate feelings in certain situations	16 (59)
Self-neglect	Had little or no interest/motivation in everyday activities, like bathing, grooming, taking care of him/herself, getting dressed, eating, etc.	14 (52)
Blunted affect	Spoke in a monotone/flat voice (did not show many changes in voice, or facial expressions)	10 (37)
Other symptoms^a^		
Agitation, irritability, hostility	Seemed like he/she was feeling agitated/irritable/hostile	22 (81)
Cognitive impairment	Had problems with memory, concentration/attention, organizing, planning	20 (74)
Anxiety	Seemed like (or reported to you) he/she was anxious or worried	18 (67)
Depression	Seemed like (or reported to you) he/she was depressed (expressed depressive thoughts/excessive sadness)	14 (52)
Worry that something is wrong with mind	Worried that something is wrong with mind	11 (41)

DSM-5 = *Diagnostic and Statistical Manual of Mental Disorders, 5th Edition*

Note: Table was organized by frequency of symptom/behavior reported

Note: Caregivers selected as many symptoms/behaviors as they thought applied

Note: Caregivers in the first two focus groups were asked to rank only the positive symptoms and exclude the negative or other symptoms in their rankings. The subsequent focus groups ranked all symptoms

^a^This category was added by the project team

### Analyses

The data analysis process was consistent across each focus group. Immediately following each focus group, the moderators and observers debriefed and recorded initial thoughts from the focus groups, including the most important concepts and dominant trends. Issues, if identified during the debriefings, were resolved between the lead group moderator and observer(s).

This step was followed by more in-depth thematic analysis, led by the lead moderator (D.B. DiBenedetti). The thematic analysis was facilitated by the raw focus group data (i.e., the moderators’ field notes and the focus group transcripts) and was conducted using Atlas.ti software. Using standard qualitative analysis and thematic coding methods (e.g., [29]), the moderators identified and summarized patterns found in the data. Dominant trends were identified in each focus group, and results were compared across subsequent groups to generate themes or patterns in participants’ responses and to evaluate the relative importance of concepts. Any discrepancies were resolved in discussion between the moderators. This process also confirmed that saturation had been reached. No formal statistical analyses or comparisons were conducted.

## Results

### Participant characteristics

A total of 27 caregivers participated across eight focus groups (Chicago [2 groups], *n* = 8; Philadelphia, *n* = 4; Raleigh [2 groups], *n* = 5; Phoenix [2 groups], *n* = 6; Los Angeles, n = 4). Most caregivers were female (78%) (Table 2). Most care recipients (individuals with TRS) were male (59%) and had been diagnosed with schizophrenia for an average of approximately 18 years; 11 individuals had been diagnosed for 10 or fewer years (Table 2).

**Table 2:** Characteristics of caregiver participants (*N* = 27) and care recipients

Characteristics	Total (*N* = 27^a^)
Caregiver characteristics	
Caregiver’s age, mean (SD)	48 (11.2)
Caregiver’s sex, *n* (%)	
Male	6 (22)
Female	21 (78)
Caregiver’s race/ethnicity, *n* (%)	
Caucasian	14 (52)
African American	10 (37)
Hispanic	1 (4)
Asian	2 (7)
Caregiver’s relationship to patient, *n* (%)	
Sibling	8 (30)
Parent	5 (19)
Adult child	3 (11)
Spouse/significant other^b^	5 (19)
Other family member	3 (11)
Friend or other	3 (11)
Caregiver’s education level, *n* (%)	
Some college	8 (30)
College degree	16 (59)
Postgraduate	3 (11)
Caregiver’s employment status, *n* (%)	
Full time	17 (63)
Part time	4 (15)
Retired	1 (4)
Unemployed	4 (15)
Student	1 (4)
Care recipients’ characteristics^c^	
Patient age, mean (SD)	46.9 (18.6)
Patient gender, *n* (%)	
Male	16 (59)
Female	11 (41)
Years since schizophrenia diagnosis, mean (SD)	18.4 (14.1)
Years since schizophrenia diagnosis, *n* (%)	
< 5 years	5 (19)
5–10 years	6 (22)
11–15 years	4 (15)
≥ 16 years	12 (44)
Patients’ living situation, *n* (%)	
With caregiver	14 (52)
With family	7 (26)
Alone	4 (15)
Other	2 (7)
Patient education level, *n* (%)	
Less than or equal to high school	15 (56)
Some college	6 (22)
College degree	6 (22)
Patient employment status, *n* (%)	
Full time	2 (7)
Part time	5 (19)
Unemployed	17 (63)
Retired	3 (11)
Comorbid psychiatric disorders,^d^ *n* (%)	
Depression	7 (26)
Anxiety	7 (26)
Bipolar	7 (26)
Major mood disorder	1 (4)
Major depressive disorder	3 (11)
Other schizophrenia-related disorders^e^	4 (15)
Patients’ comorbid somatic conditions,^f^ *n* (%)	
High blood pressure	7 (26)
Overweight	8 (30)
Diabetes	5 (19)
Current AP treatment	
AP monotherapy	20 (74%)
Atypical AP	15 (56)
Typical AP	5 (19)
AP combination therapy	7 (26%)
2 atypical APs	3 (11)
2 atypical APs and 1 typical AP	2 (7)
1 atypical AP and 1 typical AP	1 (4)
3 typical APs	1 (4)

*AP* antipsychotic medication, *SD* standard deviation

^a^One caregiver provided care for an individual whom the participant reported as having been stable for 1 year on current treatment

^b^Includes the live-in caretaker (former wife) of an individual with treatment-related schizophrenia

^c^As reported by caregivers

^d^All diagnosed before schizophrenia diagnosis. Some patients had more than one psychiatric disorder

^e^Related disorders include schizoaffective or schizophreniform disorders, schizotypal personality disorder, and brief psychotic disorder

^f^Some patients had more than one comorbid condition

At the time of screening, 20 of 27 care recipients (74%) were currently on AP monotherapy, with 15 using an atypical and 5 using a typical AP. Seven care recipients (26%) were currently taking AP combination therapy; Table 2 presents the number and type of APs received.

### Caregiving duration and responsibilities

Caregivers reported being in their role for an average of 11 years and providing direct care to an individual with TRS for an average of 36.8 h per week (Table 3). The sample included a mix of those providing care for a relatively short time (< 5 years) and those providing long-term care (≥16 years) (Table 3). Direct care hours involved a range of activities, including medical care. Most caregivers were involved in coordinating and attending care recipients’ physician appointments (*n* = 25; 93%) and managing medication (*n* = 23; 85%). Caregivers also reported providing emotional and social support (*n* = 20; 74%), assisting with activities of daily living (*n* = 15; 56%), and providing financial support or managing finances (*n* = 15; 56%). All caregivers reported being “on call” for emergencies, and 22 (81%) reported being on call for more than 100 h per week; of these, 21 caregivers (78%) reported being on call “24/7.”

**Table 3:** Time commitments of caregivers (*N* = 27)

Characteristics	Total (*N* = 27^a^)
Years in role as caregiver, mean (SD)	11 (7.5)
< 5 years	7
5 to ≤10 years	9
11 to ≤15 years	4
≥ 16 years	7
Hours providing direct care/week, mean (SD)	36.8 (21.4)
Hours “on call” for patient/week, mean (SD)^b^	147.3 (42)

*SD* standard deviation

^a^One caregiver provided care for an individual whom the participant reported as having been stable for 1 year on current treatment

^b^Eleven participants (6 in Chicago and 5 in Phoenix) during screening said “24/7” instead of exact number of hours on call. In Raleigh and Los Angeles, participants that reported “24” hours were referring to “24 h per day.” Averages were calculated by converting the 24/7 and 24 to 168 h

Several caregivers reported that providing emotional care was the most challenging of the care types they provided. Providing emotional care was taxing and sometimes burdensome for caregivers, as it seemed to be an unrelenting task associated with their role:

*“The emotional aspect of the caretaking for me is the most pronounced; the activities themselves are not that difficult to do. The emotional aspects…can feel like a burden.”*

*“The emotional [care]…It’s very draining…She’s your mom…the roles have totally reversed. I’m on the phone with her for hours and hours trying to convince her not to just pick up and move wherever because she got a weird look at the store.”*

### Persistent schizophrenia symptoms

Table 1 presents the list of positive symptoms, negative symptoms, and other symptoms assessed and their non-clinical descriptions as provided to caregivers. Of the 30 individual symptoms/behaviors evaluated, 19 were reported by more than half of the caregivers as those their care recipients exhibited or reported (Table 1). While there was no symptom or behavior that all caregivers selected, several were reported by most caregivers, including auditory hallucinations (*n* = 24; 89%); agitation, irritability, hostility (*n* = 22; 81%); suspiciousness (*n* = 21; 78%); tangentiality (*n* = 20; 74%); and cognitive impairment (*n* = 20; 74%).

Participants then ranked the category of symptoms/behaviors that they found the most challenging as caregivers (Table 4). Positive symptom categories were most commonly reported to be the most challenging—particularly suspiciousness/persecution (*n* = 19; 70%), disorganized speech (*n* = 11; 41%), and delusions (*n* = 10; 37%).

**Table 4:** Rankings of most challenging schizophrenia-related symptoms/behaviors (*N* = 27)

	Ranked #1, *n* (%)	Ranked #2, *n* (%)	Ranked #3, *n* (%)	Total, *n* (%)
Hallucinations	3 (11)	4 (15)	2 (7)	9 (33)
Delusions	3 (11)	4 (15)	3 (11)	10 (37)
Disorganized speech	4 (15)	3 (11)	4 (15)	11 (41)
Suspiciousness/persecution	5 (19)	6 (22)	8 (30)	19 (70)
Negative symptoms^a^	3 (11)	1 (4)	5 (19)	9 (33)
Other symptoms^a^	4 (15)	4 (15)	0 (0)	8 (30)

Note: Percentages were calculated as n divided by the total number of participants (*N* = 27)

^a^In the first two focus groups, caregivers were asked specifically to rank only the four positive symptoms (either the top-level headers or the individual symptoms/behaviors). Ranking of all six categories (top-level headers only) was allowed for all subsequent groups

Caregivers described significant challenges associated with their care recipients’ persistent symptoms and behaviors.

*“He constantly feels like people are watching him or is suspicious of people. That’s very frustrating when we go out in public together. He feels that people in helicopters are following him. And so at night, [since] there’s obviously a lot of…air activity all the time…we have to usually be home before sunset.”*

Caregivers also described care recipients’ disorganized speech and thought patterns.

*“As far as the rambling and not being able to hold a topic, it impedes your communication with them and that kind of leaves you out. You can’t really pull them in if you don’t understand [them] or they can’t express it.”*

Agitation, irritability, and/or hostility was the most frequently reported symptom/behavior in the other symptom category across the eight focus groups.

*“The feeling agitated, irritable, and hostile…you never know what you’re coming into. When I visit her, I never know if she’s going to be agitated and hostile.”*

*“I’ll leave work and go and see what’s going on with her. Sometimes she’ll let me in, sometimes she won’t. I do have a key. Sometimes when I come in, she’s really hostile, she’s cursing.”*

### Impact of persistent schizophrenia symptoms and behaviors on caregivers

All 27 caregivers noted that caring for someone with TRS negatively impacted some aspect of their lives, particularly their social lives (*n* = 24; 89%) and mental health (*n* = 23; 85%). Some described a feeling of stigma associated with schizophrenia:

*“I think we go out a little less because he is so hard to get out of the house…So we don’t go out as much as I would like to. And we certainly don’t go to as many public places that I would like to. I used to really enjoy going out and having friends. But it just became such an issue because when I got home, he didn’t understand where I was and he would get so paranoid.”*

*“I have no friends. My mom has no friends. I’ve always felt stigmatized…I was always ashamed, I never wanted anybody to know [mom had schizophrenia]. As a result, I had very little friends growing up because even if I tried to establish relationships, my mom would do something ‘crazy,’ then they would no longer want to be my friend.”*

Some caregivers also reported that family relationships and romantic relationships were negatively affected by their caring for an individual with TRS. Several caregivers had “given up” on dating altogether because of the challenges their caregiving role presented when trying to date or meet a potential partner, while caregivers currently in relationships noted how caring for an individual with TRS had negatively impacted relationships with their spouses or significant others.

*“I can’t be married under these situations…He maxed his credit cards out. Legal problems…And he lost his job. I mean, everything was crashing in on me...I couldn’t absorb all of that. So my parents made me divorce him.”*

*“I had to give up dating for a lot of reasons…It was easier to stop dating because of the fact the situation is so unusual and he certainly didn’t make it easier.”*

*“I’d been taking care of him for a few years before he was diagnosed…So we were divorced and he lived with my wife until he was 18 and then he came back to live with me because she couldn’t handle it.”*

Several caregivers (*n* = 6; 22%) noted that they were taking antidepressants or anxiolytics because of their role as a caregiver for someone with TRS.

*“Honestly, I had to start taking medication for depression, too. It just got to be a little too much.”*

Many caregivers (*n* = 17; 63%) discussed how their physical health was negatively affected by caring for an individual with TRS, including impacts on sleep and self-care. A few even noted more significant medical problems that they attributed to the stress of caring for an individual with TRS.

*“I don’t sleep much…I need to be up when she’s up, for the most part. It’s a trust issue. I don’t know what the voices are going to tell her to do.”*

*“I had a stroke about a year and a half ago. And I still think the stress [of caregiving] caused the stroke.”*

Over three-quarters of the caregivers (*n* = 21; 78%) indicated that their finances, including employment, professional opportunities, savings, ability to travel, and future financial plans, were impacted by their role in caring for an individual with TRS. Some caregivers reported that the demands of caregiving led them to take additional time off work, cut back on their hours, refuse promotions, and take on less responsible roles.

*“So I, many times, have to…just clean up the mess…I spend money on him…Be it basic necessities…phone bills and lawyers.”*

*“I was teaching at one point in time…[I] no longer teach anymore because he was having so many frequent incidents.”*

*“I [cannot] retire early or soon. I’m going to [work longer to] make more money so then I can take him [brother] with me.”*

### Impact of persistent schizophrenia symptoms on care recipients’ and caregivers’ safety

Caregivers expressed concern for the safety of their care recipients. Safety concerns were related to the care recipients’ persistent symptoms/behaviors, as well as worries that they might be victimized by others.

*“We keep him inside. We keep him safe…But he started going across the street to see some neighbors when I was at work. And they robbed him.”*

*“Sometimes when we’ve gotten stopped by the police, I’m very afraid, because they think he’s drugged or they think he is high or drunk because of the way he speaks.”*

*“He’s very naive, and people prey upon him and take advantage of him.”*

Hostility and the potential for personal violence were concerns, as caregivers expressed worry that their care recipient might harm them, themselves, or others due to ineffective AP medication. Safety concerns were most commonly mentioned in association with symptoms of agitation, irritability, or hostility, reported by 22 caregivers (81%).

*“She [patient] would never really want to [hurt] intentionally. But once she reaches that level of frustration to where it becomes physical and it’s like Tasmanian Devil type tornado, you can get injured. Anything could get damaged or broken.”*

*“I’m starting to get concerned with that because he talks about like buying a firearm, learning martial arts, and I’m concerned for that.”*

*“If he’s acting [out], call the police. Don’t hesitate [to call the police]. They want me to control him. I can’t control him when he gets like that. I can’t do the things that the police could do. I’d get arrested if I do. So, yeah, he can be dangerous.”*

Caregivers also discussed the impact of TRS on the lives of their care recipients. They expressed that TRS was challenging and burdensome for their care recipients, particularly after years of having medications fail to improve their symptoms and behaviors.

*“I thought that once he got his medication, he would be fine because he always had the desire to want to finish college. He only had 1 more year…It’s like, ‘Lord, when is it going to stop? When is it going to end? When is it going to be where he can function on his own?’ I’m not promised to be here forever; it’s like I always think, ‘What’s going to happen to my son if something was to happen to me?’”*

### Medication experiences

Caregivers described their care recipients’ responses to medications for schizophrenia. Specifically, caregivers were asked if previous medications seemed to work initially but then stopped working with time or if they did not seem to work at all. A small number of caregivers (*n* = 4, 15%) noted that their care recipients had never responded to APs; most reported that their care recipients initially responded to treatment (even if only partially), but eventually stopped responding.

*“It doesn’t matter what medication she’s on, and very few of them have ever worked for her for any period of time. They might start out good but then after a while, they don’t work.”*

Caregivers also noted that their care recipients’ trying different medications over time with little to no symptom improvement took an emotional toll on both the caregiver and recipient. Caregivers perceived that their care recipients were frustrated after spending years trying different medications, only to have them prove ineffective at resolving the symptoms and behaviors that made them unable to live like others. When asked about the experience of having multiple medications fail, caregivers described frustrations and differing perspectives on their hope for treatments working for their care recipients.

*“It’s seeing that person crying for help, asking you that they want to live normal. They’re worried that something’s wrong with them, that they’re never going to be [normal] and seeing them not wanting to take any medication, because they already know it’s not going to do anything.”*

*“[I feel] helpless when a medication doesn’t work or the symptoms don’t stop just because he has to take the medication.”*

Finally, caregivers were asked how they thought their lives would be positively impacted if their care recipient’s symptoms/behaviors were improved by a new medication. Although this question was difficult for some to even contemplate, across all focus groups, decreased worry and more freedom for themselves and their care recipients, was commonly reported.

*“Freedom. I guess that’s the word, freedom, personal freedom.”*

*“[Without husband’s symptoms] I could keep the TVs plugged in. His phone would not get dismantled on a nightly basis…it would feel like life would resume.”*

*“Maybe she would be able to enjoy her life because she’s not able to enjoy her life now.”*

*“She could do what she always wanted to do and get back to college and make a difference in the world.”*

## Discussion

This study aimed to characterize the experiences, burden, and perspectives on AP treatments of caregivers for individuals with TRS who, unlike treatment-responsive patients, persistently experience moderate-to-severe positive symptoms despite treatment. To our knowledge, this is the first study to explore the experiences and perspectives of caregivers of individuals who have no history of sustained response to antipsychotic medication. On average, caregivers in this group had been providing care for 11 years and care recipients had been diagnosed with schizophrenia 18 years before the study. For some, the difference between years since diagnosis and years of care provision was because others had provided care before the current caregiver stepped in. Other caregivers indicated that their care recipients had responded to treatment initially but eventually developed TRS, thus requiring additional care later in their disease course. Nonetheless, the results of this study reflect the experiences of people who had provided care for loved ones with persistent symptoms for many years. It is unknown whether and how many caregivers eventually cease providing care for individuals with TRS or the reasons for discontinuing care. Research is needed to explore the perspectives of caregivers of persons with TRS throughout the disease course to understand the significant impact of TRS on individuals with TRS, their families, friends, and other caregivers.

Caregivers in our sample reported spending an average of 37 h weekly providing direct care for their loved ones with persistent schizophrenia symptoms—the equivalent of a full-time job, and more than estimates from previous survey studies that caregivers of individuals with severe mental illness spend 22 h providing informal care each week [25, 26]. Previous research has shown that caregivers of individuals with mental illness may underestimate their caregiving time due to recall bias [25]; thus, caregivers in this study in fact may spend more time providing care than they estimated. Caregivers were commonly involved in attending physician or therapist appointments and managing medication, and their close involvement in their care recipients’ medical care suggests that care strategies for individuals with TRS should consider how best to target those in important informal caregiving roles. All caregivers reported being “on call” for emergencies, with nearly 80% being on call “24/7.” Other types of care provided included offering regular emotional and social support, assisting with activities of daily living, and providing financial support. Some described a stigma associated with schizophrenia, which previous research has shown negatively affects quality of life for both individuals with schizophrenia and their caregivers [30, 31]. Caregivers noted that being on call and providing emotional support were more difficult for them than providing direct care.

Caregivers reported that their care recipients exhibited many persistent positive symptoms, negative symptoms, and other symptoms of schizophrenia, despite current AP treatment. Thus, caregiver participants’ experiences are likely to differ from those providing care for individuals with treatment-responsive schizophrenia, who have periods with less-severe or no symptoms. Notably, the most frequently reported symptoms/behaviors (e.g., auditory hallucinations) were not the most challenging for caregivers; in fact, nearly three-quarters of caregivers (70%) identified suspiciousness/persecution as the most challenging symptom category. The potential downstream effects of persistent positive symptoms such as hostility, aggressiveness, and irritability warrant additional investigation.

All caregivers reported that caring for an individual with TRS had a significant impact on their personal lives, especially their own mental health, thus suggesting that mental/emotional strain may be one of the most significant impacts of providing care. For most, the emotional toll of their responsibilities led to feelings of being overwhelmed, stressed, drained, burdened, frustrated, angry, depressed, and/or anxious. Other areas of great impact were caregivers’ social lives and romantic relationships; some participants reported abandoning dating or ending marriages and feeling socially isolated. The combination of persistent positive symptoms and ineffective medications resulted in caregivers’ having to spend significant time with or on call for their care recipients, rendering social activities and significant relationships difficult. Caregivers’ professional lives were also affected, potentially leading to considerable loss of future income. Moreover, many participants, particularly primary caregivers, also expressed significant concern for the care recipient’s future.

These findings echo those from a multinational study in which caregivers of individuals with schizophrenia reported experiencing worse quality of life as a consequence of caregiving and greater direct and indirect costs than caregivers of individuals with Alzheimer’s disease, cancer, or stroke in the areas of mental health, emotional health, and social functioning [20]. The burden of caregiving was found to increase with worsening schizophrenia symptoms [20, 32]. Schizophrenia caregivers also commonly experience physical health impacts including stress-related comorbidities including insomnia, pain, headaches, heartburn, anxiety, and depression [20, 33].

People with schizophrenia are more likely to engage in violent behavior than those without the condition [34]. Nevertheless, violent behavior exhibited by individuals with schizophrenia remains relatively rare, with estimates ranging from 8 to 30% [35–38]. Female family members, particularly mothers, may more often be the targets of these rare acts of serious violence than others [39]. High positive symptom burden (> 3 symptoms), medication non-adherence, substance abuse, impulsivity, and a history of violence are important factors associated with hostility and aggression, which also occur more frequently in younger males than in others with schizophrenia [1, 38, 40]. Addressing and treating hostility/aggression in individuals who do exhibit these behaviors is a clinical imperative and an important consideration in TRS [41, 42]. In the current study, caregivers reporting hostility/aggression in their care recipients noted that this behavior was largely due to persistent paranoia causing patients to act out. At the same time, people with schizophrenia are far more likely to be victims of violence than to initiate violence [19, 43]. Caregivers in this study reported fearing for their care recipients’ safety because of their unpredictable behavior. Further research is warranted on how persistent positive symptoms affect caregivers’ sense of safety for themselves and others, and how this may influence the care provided.

Many caregivers described frustration and other emotional reactions associated with persistent moderate-to-severe positive symptoms resulting from continuing AP medication failure and questioned whether their care recipients would ever find relief. Caregivers’ expectations of new medications for TRS were mixed, with some noting that they had little hope that new medications would be different than those currently available. Regardless of caregivers’ expectations of new medications, an overarching theme that emerged was freedom. Some caregivers perceived that if they no longer had the responsibility of caring for an individual with TRS (e.g., because of a “cure,” or amelioration of challenging symptoms), both the caregiver and their loved one would have the freedom to return to work/school, travel, socialize, and enjoy a better quality of life.

The important role of caregivers in shared decision-making for psychiatric medication management has been acknowledged [44]. In the current study, caregivers described their experiences with their care recipients’ schizophrenia treatment, but not all caregivers had an opportunity to participate in treatment decisions. Future research should explore how informal caregivers can be optimally involved in treatment decisions for individuals with TRS.

The experiences of caregivers of people with TRS as found in our study likely differ from those of caregivers of individuals with treatment-responsive schizophrenia. Caregivers of individuals with TRS continually deal with high levels of persistent symptoms over long periods of time, leading to substantial burden and, for many, feelings of hopelessness. Furthermore, compared with caregivers of individuals with treatment-responsive schizophrenia, who may find relief during periods of patients’ symptom remission, caregivers’ responsibilities in TRS are unrelenting. Prior research has emphasized a need for support and relief for informal caregivers of individuals with mental disorders [20, 25, 26]. Given the considerable burden of providing care for individuals with TRS that the current study identified, there is a critical need for practical, social, and emotional support and relief for this caregiver population. It is important that local and federal health policy makers and funding agencies be aware of the unique needs of the community and families created by people with this chronic form of schizophrenia.

Several limitations of this study must be noted. Caregivers reported during screening that their care recipients had failed ≥2 APs and had ≥2 moderate positive symptoms; however, these data were self-reported and not clinician confirmed. As with most qualitative studies, the participants in these focus groups may not represent a broader TRS sample, potentially limiting generalizability. Nonetheless, the sample size used in the current study is consistent with sample sizes from other qualitative studies of perceptions of caregivers for individuals with schizophrenia [45, 46] and was sufficient to address the study objectives. Furthermore, participants were drawn from five geographically diverse US locations, and their feedback across locations suggested saturation.

Caregivers of individuals whose TRS was currently being treated with clozapine were ineligible for this study. Clozapine is the only medication currently indicated for TRS, yet it is often delayed or underutilized due to various barriers including monitoring requirements and the potential for serious adverse events [4, 12, 47]. Should these barriers be overcome and effective treatment be provided earlier in the disease course, consistent with international treatment guidelines [9–11], the likely result would be improved clinical patient outcomes, with fewer demands and less emotional stress for caregivers. A question for future research is whether caregivers of individuals receiving clozapine for TRS report different perspectives and experiences than those captured in this study.

## Conclusions

The results of this qualitative research detail the significant clinical, humanistic, economic, and societal impact of caring for individuals with TRS, highlighting a critical gap and a need for additional research. Caregivers provided compelling reports regarding the destructive impact on their lives when persistent positive symptoms, including suspiciousness/persecution, hallucinations, and disorganized speech and other symptoms of schizophrenia, such as agitation, irritability, and hostility, were not adequately controlled by existing medications. Means of providing practical, social, and emotional support for caregivers of individuals with TRS are needed, and future research should explore how informal caregivers can be optimally involved in treatment decision making. In addition, there is a critical need for effective and tolerable treatments for TRS that will control patients’ symptoms and improve psychosocial functioning, productivity, and quality of life for individuals with TRS and their caregivers.

## Abbreviations

AP: antipsychotic
SD: standard deviation
TRS: treatment-resistant schizophrenia
US: United States
